# Variable directionality of gene expression changes across generations does not constitute negative evidence of epigenetic inheritance

**DOI:** 10.1093/eep/dvv005

**Published:** 2015-10-20

**Authors:** Abhay Sharma

**Affiliations:** CSIR-Institute of Genomics and Integrative Biology, Council of Scientific and Industrial Research, Sukhdev Vihar, Mathura Road, New Delhi 110025, India

**Keywords:** epigenetic inheritance, endocrine disrupting chemicals, gene expression, transcriptome, vinclozolin, di-(2-ethylhexyl)phthalate

## Abstract

Transgenerational epigenetic inheritance in mammals has been controversial due to inherent difficulties in its experimental demonstration. A recent report has, however, opened a new front in the ongoing debate by claiming that endocrine disrupting chemicals, contrary to previous findings, do not cause effects across generations. This claim is based on the observation that gene expression changes induced by these chemicals in the exposed and unexposed generations are mainly in the opposite direction. This analysis shows that the pattern of gene expression reported in the two generations is not expected by chance and is suggestive of transmission across generations. A meta-analysis of diverse data sets related to endocrine disruptor-induced transgenerational gene expression alterations, including the data provided in the said report, further suggests that effects of endocrine disrupting chemicals persist in unexposed generations. Based on the prior evidence of phenotypic variability and gene expression alterations in opposite direction between generations, it is argued here that calling evidence of mismatched directionality in gene expression in experiments testing potential of environmental agents in inducing epigenetic inheritance of phenotypic traits as negative is untenable. This is expected to settle the newly raised doubts over epigenetic inheritance in mammals.

## Introduction

Transgenerational epigenetic inheritance in mammals is highly controversial and a subject of intense debate [1–9]. Its existence has been questioned due to inherent difficulties in experimental demonstration of germline transmission of environmentally induced characters [1,4,5,8–11]. However, a recent report [12] has opened a new front in the ongoing debate by claiming, contrary to evidence presented in several studies published previously [13–21], that endocrine disrupting chemicals (EDCs) do not cause transgenerational effects. The supposed negative evidence has been highlighted [22] as crucial in refuting epigenetic inheritance in mammals. To avoid any confusion that may further add to the existing controversies, it is necessary to revisit the original findings [12] underlying the conclusion drawn, that EDCs do not cause transgenerational effects. Notably, a reassessment of the reported data [12] presented here provides evidence that supports, not refutes, epigenetic inheritance. In essence, the authors of the published report [12] presumed that, to call EDCs effects as potentially transgenerational, the chemical induced changes in expression of genes must be in the same direction in both exposed and unexposed generations. Here, it is shown that, irrespective of direction, the pattern of altered gene expression observed in the two generations is not expected by chance and is suggestive of EDCs' intergenerational effects. Additional analysis is presented to further suggest that negative evidence of epigenetic inheritance based strictly on directionality of phenotypic change [12] is not tenable. Altogether, this article addresses the recent doubts created by the so-called negative evidence of the potential of EDCs in inducing transgenerational effects in mammals.

## The Supposed Negative Evidence

In brief, the authors of the said report [12], Iqbal *et al*., treated G0 female mice with the EDC vinclozolin (VZ), di-(2-ethylhexyl)phthalate (DEPH), or bisphenol A during the time when the germ cells of the G1 male fetus undergo global *de novo* DNA methylation and imprint establishment and examined differentially methylated regions and allele-specific transcription of imprinted genes in multiple generations, for investigating if environmental effects are inherited. After finding no evidence of epigenetic inheritance in this candidate approach, the authors carried out genome-wide DNA methylation and transcriptomic analyses of the purified G1 and G2 prospermatogonia, termed reprogrammed G1 (G1R) and G2 (G2R) hereafter, to further investigate potential inheritance of EDC-induced effects. In this unbiased approach, Iqbal *et al*. found methylomic and transcriptomic changes in both G1R and G2R. Overall, these changes were, however, not in the same direction across generations, against the criteria set forth by the authors for calling epigenetic inheritance. Although negative findings in methylation analysis do not completely exclude the possibility of epigenetic inheritance since other epigenetic factors like histone marks and RNA may also potentially mediate germline inheritance, Iqbal *et al*. did find the lack of persistent transcriptomic changes, because epigenetic modifications are likely manifest in aberrant gene expression, as conclusive evidence for refuting epigenetic inheritance. The authors found differential expression of 325 genes in common between DEPH-associated G1R and G2R and 284 genes in common between VZ-associated G1R and G2R. The authors split the data into up- and down-regulated genes and found that a significantly greater number of the common changes occurred in the opposite direction between generations. This prompted them to conclude that EDC effects are not inherited.

## Data Reassessment Suggesting Positive Evidence

**Figure 1:**
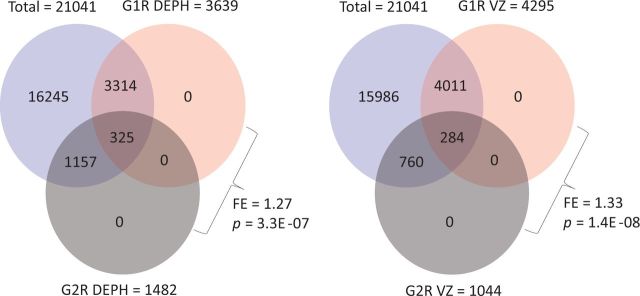
primary within-study support for EDCs' effects across generations. The overlaps of differentially expressed genes between G1R and G2R are shown for DEPH and VZ. Note statistically significant overlap in sets of genes identified with altered expression following treatment with either of the two EDs. FE, fold enrichment. *P* indicates hypergeometric distribution probability. The *P* values shown relate to the probability of obtaining the exact number of overlaps, not the probability of at least that many overlaps. It is, however, notable that the overlaps remain highly significant even when the probability of at least that many overlaps is applied. The data provided in Table 4 and Supplementary File S12 of the report by Iqbal *et al*. [12] were used for calculating fold enrichment and hypergeometric distribution probability

To examine epigenetic inheritance, the probability of finding 325 common differentially expressed genes between DEPH-associated G1R and G2R, and 284 genes between VZ-associated G1R and G2R should have been tested in the first place. Considering the RefSeq gene count for the Affymetrix 1.0 ST array used in the transcriptomic analysis by Iqbal *et al*. as 21 041 [23], the set of differentially expressed genes in one generation was found to be significantly overrepresented in that of the other, for both DEPH and VZ treatment groups (Fig. 1). Regarding expression change in the opposite direction across generations, the observation that compelled Iqbal *et al*. to refute epigenetic inheritance, it does not necessarily compromise the positive evidence of transgenerational effects obtained above because phenotypic variability across generations is known in epigenetic inheritance. For example, transgenerational weakening and strengthening of phenotypes have been reported in several studies in mammals [24–33]. Moreover, gene expression changes in opposite direction across generations have also been reported in studies pertaining to effects of environmental agents in animals [34,35]. In mice, it has been reported that exposure of F0 pregnant females to VZ causes downregulation of *Lin28a*, one of the two paralogs encoding a microRNA binding protein, in primordial germ cells (PGCs) from F1 and F2 embryos, and, in a rebound-like response, a slight upregulation of the paralog in PGCs from F3 embryo [34]. Similarly, the expression levels of the other paralog, *Lin28b*, were found to be decreased in PGCs from F1 and F2 animals, whereas the levels tended to become normalized in F3 individuals [34]. In another example, nicotine exposure in the worm *C. elegans* in F0 generation has been found to result in altered expression of various miRNAs in the exposed and the unexposed F1 and F2 generations, with several miRNAs showing expression changes in opposite direction between generations [35]. 

## Additional Evidence Supporting Positive Evidence for Transgenerational Epigenetic Inheritance

Iqbal *et al*. [12] also did not consider in their transcriptomic analysis, unlike DNA methylation analysis that they performed, comparison with previously reported gene expression data related to EDCs' transgenerational effects. Transcriptomic profiles representing various embryonic and adult tissues from multiple generations of rats [13–21], investigated for transgenerational effects of VZ, have been used previously [36,37] for gene enrichment analysis in epigenetic inheritance. In the reported investigations [13–21], F0 females were exposed to VZ during gestation and the resulting unexposed generations from F1 to F3 examined for transcriptomic alterations. To address the deficiency in the report in question [12], the compiled sets (Supplementary Table S1) of differentially expressed genes identified by the original authors [13–21] in the aforementioned studies (Supplementary Table S2) and used in a subsequent meta-analysis [36,37] were compared with 284 and 325 differentially expressed genes identified by Iqbal *et al*. [12] following VZ and DEPH treatment, in that order. As such, the gene lists used in these comparisons were irrespective of directionality of expression changes, with up- and down-regulated genes combined together for each group. In this analysis of mouse and rat gene expression data from diverse studies, the entire set of human genes listed in the gene ontology [38] was used for normalization, to find overlap between previous gene sets and genes identified by Iqbal *et al*. The premise of this analysis was that a higher fold enrichment with greater statistical significance in VZ-VZ comparison relative to VZ-DEPH comparison would indicate epigenetic inheritance.

Hypergeometric distribution probability test was performed to examine enrichment. For this, all the human genes in gene ontology [38] were considered as the population size, genes that were identified by Iqbal *et al*. [12] as differentially expressed following VZ or DEPH treatment and present in gene ontology as the population successes, and genes that were identified previously as differentially expressed following VZ treatment [13–21] and present in gene ontology as the sample size. The overlaps between the population successes and the sample size were obtained as sample successes. A nominal *P*-value cutoff of 0.05 was used in the enrichment analysis.

**Figure 2:**
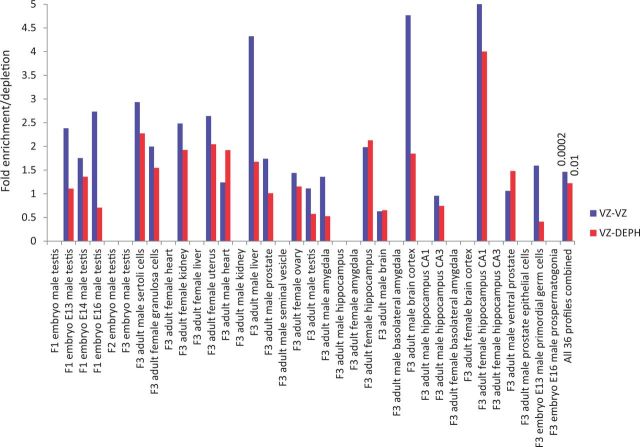
meta-analysis supporting EDCs' effects across generations. The overlaps of differentially expressed genes between previously identified sets (Supplementary Table S1) in transgenerational experiments investigating effects of VZ, and the common G1R and G2R genes shown in Figure 1 for DEPH and VZ are depicted. The last pair of bars show combined analysis. Numbers above the bars indicate hypergeometric distribution probability. Missing bars indicate insufficient data for statistical analysis, due to nil or less than 5 overlapping genes between two sets. Note a general trend for higher fold enrichment for VZ-VZ overlaps across studies, compared to VZ-DEPH overlaps. Also note a greatly significant overlap for VZ-VZ comparison, relative to VZ-DEPH comparison, in the combined analysis. The details of studies representing figure labels in the *x*-axis are provided in Supplementary Table S2

Importantly, a higher enrichment with greater significance is indeed observed for VZ-VZ overlap than VZ-DEPH overlap (Fig. 2). A lower overlap between VZ and DEPH is not surprising because being EDCs both the compounds are expected to cause gene expression alterations that are similar to a certain extent. Together, the results of the meta-analysis are consistent with the above evidence (Fig. 1) that the data obtained by Iqbal *et al*. [12] support, not refute, the potential of EDCs in causing altered gene expression across generations. 

## Conclusion

Contrary to the conclusion of Iqbal *et al*. [12] that EDC-induced effects do not persist in the unexposed generation, reassessment of their gene expression data suggests the opposite; the effects seem to be transmitted across generations through the germline. Clearly, a statistically significant overrepresentation of genes differentially expressed in EDC exposed generation in the next unexposed generation is consistent with epigenetic inheritance. Meta-analysis of diverse data sets related to EDC's transgenerational effects on gene expression further supports this. Phenotypic variability in epigenetic inheritance is known and is consistent with the recently proposed concept of transgenerational systems biology [7–9] that hypothesizes a role of gene networks, besides DNA methylation, histone modifications and RNA, in epigenetic inheritance. Given the far reaching implications of epigenetic inheritance in human health and evolution, future experiments need to focus on the molecular mechanisms underlying inheritance of acquired traits.

## Supplementary Material

Supplementary Data Supplementary Data
